# Regional Differences in Neuroinflammation-Associated Gene Expression in the Brain of Sporadic Creutzfeldt–Jakob Disease Patients

**DOI:** 10.3390/ijms22010140

**Published:** 2020-12-25

**Authors:** Aušrinė Areškevičiūtė, Thomas Litman, Helle Broholm, Linea C. Melchior, Pia R. Nielsen, Alison Green, Jens O. Eriksen, Colin Smith, Eva L. Lund

**Affiliations:** 1 Department of Pathology, Danish Reference Center for Prion Diseases, Copenhagen University Hospital, 2100 Copenhagen, Denmark; helle.broholm@regionh.dk (H.B.); Linea.Cecilie.Melchior@regionh.dk (L.C.M.); Eva.Loebner.Lund@regionh.dk (E.L.L.); 2 Department of Immunology and Microbiology, University of Copenhagen, 2200 Copenhagen, Denmark; tlitman@sund.ku.dk; 3 Department of Surgical Pathology, Zealand University Hospital, 4000 Roskilde, Denmark; pirn@regionsjaelland.dk (P.R.N.); jeoer@regionsjaelland.dk (J.O.E.); 4 National Creutzfeldt–Jakob Disease Research and Surveillance Unit, University of Edinburgh, Edinburgh EH8 9AB, UK; alison.green@ed.ac.uk (A.G.); Col.Smith@ed.ac.uk (C.S.)

**Keywords:** prion disease, Creutzfeldt–Jakob disease, neuroinflammation, microglia, immune response, gene expression analysis, brain microenvironment, regional pathogenesis, biomarkers

## Abstract

Neuroinflammation is an essential part of neurodegeneration. Yet, the current understanding of neuroinflammation-associated molecular events in distinct brain regions of prion disease patients is insufficient to lay the ground for effective treatment strategies targeting this complex neuropathological process. To address this problem, we analyzed the expression of 800 neuroinflammation-associated genes to create a profile of biological processes taking place in the frontal cortex and cerebellum of patients who suffered from sporadic Creutzfeldt–Jakob disease. The analysis was performed using NanoString nCounter technology with human neuroinflammation panel+. The observed gene expression patterns were regionally and sub-regionally distinct, suggesting a variable neuroinflammatory response. Interestingly, the observed differences could not be explained by the molecular subtypes of sporadic Creutzfeldt–Jakob disease. Furthermore, analyses of canonical pathways and upstream regulators based on differentially expressed genes indicated an overlap between biological processes taking place in different brain regions. This suggests that even smaller-scale spatial data reflecting subtle changes in brain cells’ functional heterogeneity and their immediate pathologic microenvironments are needed to explain the observed differential gene expression in a greater detail.

## 1. Introduction

Prion diseases, which are caused by misfolded cellular prion proteins termed prions and denoted PrP^Sc^, are a group of infectious and invariably fatal neurodegenerative diseases that can be acquired (<1%), genetic (∼15%), and sporadic (85%) [1,2,3,4]. Sporadic Creutzfeldt–Jakob disease (sCJD), the most common of all PrP^Sc^ prion diseases, has 14 subtypes that are defined by the methionine/valine polymorphism at codon 129 in the prion protein gene (*PRNP*), a type of present protease K-resistant PrP^Sc^ fragments (type 1 = 21 kDa, type 2 = 19 kDa), and distinct phenotypic features such as the appearance and distribution of Kuru plaques and pronounced cortical or thalamic changes [5,6,7,8,9].

The templated misfolding of cellular prion protein (PrP^C^) into PrP^Sc^ is central to the disease pathogenesis [10]. However, neurodegeneration is a result of multiple complex molecular processes occurring in different brain regions simultaneously. In sCJD, a highly variable selective regional vulnerability, although poorly understood, can be observed in different disease subtypes [11,12,13].

It has become clear that several brain region-specific factors must play a role. Specific cell types, with their intrinsic firing properties and metabolic activity, varying concentrations of metal ions, and differences in toxicity restricting molecular machines such as protein-folding and quality-control systems, are considered to be involved in region selection for the establishment of neurodegeneration [12].

As has been shown by many studies of prion disease animal models, neuroinflammation is an inseparable part of neurodegeneration [14,15,16]. Several studies investigating gene expression in various prion disease models indicate that upregulated genes are predominantly expressed by activated microglia, suggesting their major role in the regulation of inflammation, metabolism, respiratory stress, and possibly other functions [17,18,19].

Microglia are brain-resident macrophages constantly surveilling their microenvironment to ensure the elimination of brain cell function-disturbing events [20]. Upon the detection of abnormal activity, depending on the stimuli, microglia initiate different responses to stabilize the microenvironment [21].

Accumulating evidence suggests that microglia also play a role in synaptic organization, the control of neuronal excitability, phagocytic debris removal, trophic support for brain protection and repair, the induction of neurotoxic reactive astrocyteskilling neurons and mature oligodendrocytes [19,22,23].

However, surprisingly little is known about regional microglia-induced neuroinflammation patterns even in the most common human prion diseases [24,25,26]. Understanding the differences in potentially pathogenic molecular events taking place in different brain regions is essential for the further deciphering of complex molecular networks, their interactions, and their potential use for novel therapeutic strategies.

Thus, the current study was designed to provide a comprehensive overview of the regional neuroinflammatory differences in sCJD brain samples from deceased patients using an RNA amplification-free approach that allowed direct counting of present in the sample RNAs of interest. RNA amplification-free solution was provided by NanoString technologies, which also offered a 770-gene panel designed to represent the core processes of neuroinflammation—namely, immunity and inflammation, neurobiology and neuropathology, and metabolism and stress [27].

## 2. Results

### 2.1. Regional Differences in Gene Expression

**Figure 1:**
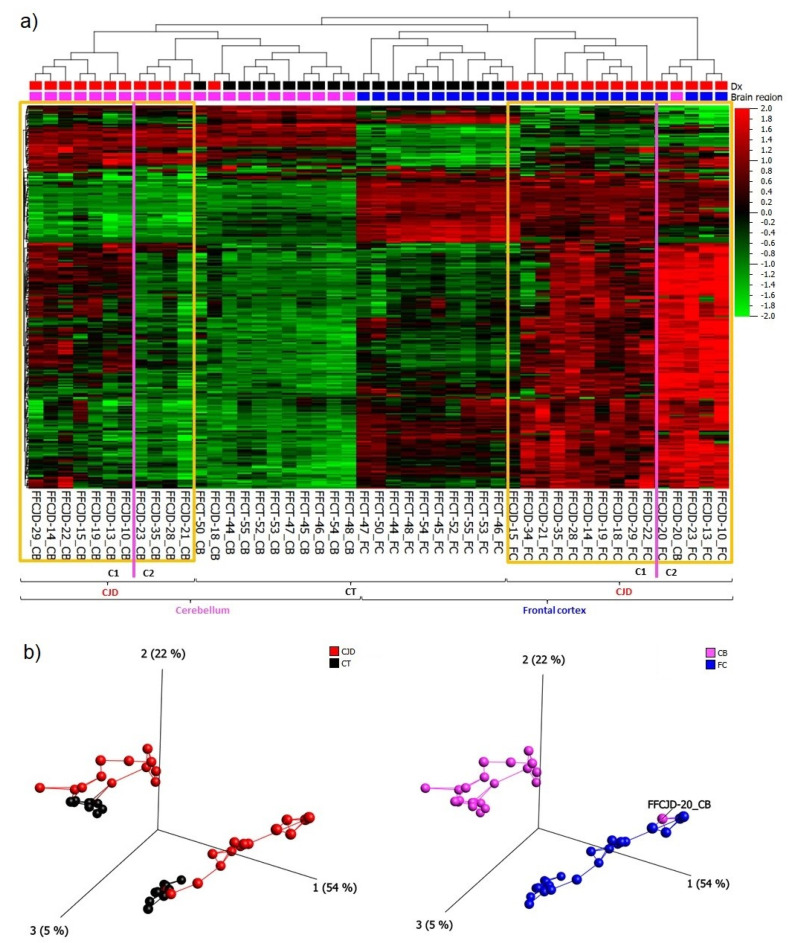
(**a**) A heatmap and unsupervised two-way hierarchical clustering based on the 265 most variable genes indicate several clusters and sub-clusters: yellow frames— sporadic Creutzfeldt–Jakob disease (sCJD) separation from control tissues (CT) and sCJD frontal cortex (FC) separation from cerebellum (CB); magenta lines—sub-clusters within sCJD FC and CB samples. (**b**) Principal component analysis indicating a clear separation between CJD and CT clusters, as well as FC and CB clusters; C1—sub-cluster 1; C2—sub-cluster 2; Dx—diagnosis.

Based on the 265 most variably expressed genes across all the samples included in the study (Supplementary material S1a, a list of the 265 genes), the following main patterns were observed: (1) primary separation between the frontal cortex (FC) and cerebellum (CB) samples, and (2) secondary separation of sCJD from control tissue samples (Figure 1a,b).

The gene expression signature of sCJD CB sample FFCJD_CB-20 suggested that it was an FC sample, and thus it was removed from further analyses. Due to major differences in gene expression profiles between FC and CB, as illustrated in the heatmap (Figure 1a), these tissues were treated separately in the subsequent analyses.

### 2.2. Top Differentially Expressed Genes (DEGs)

The neuroinflammatory gene analysis revealed several significantly differentially expressed and functionally interesting genes. We were most interested in identifying Differentially Expressed Genes (DEGs) that would be (1) common to both brain regions in sCJD samples as compared to controls; (2) unique to each brain region in sCJD samples as compared to controls; and (3) unique to a brain region regardless of sample type—i.e., sCJD or control. The three groups of DEGs identified following these comparison criteria were named as follows: (1) disease-specific, brain region non-exclusive DEGs; (2) disease-specific, brain region exclusive DEGs; and (3) disease non-specific, brain region exclusive DEGs. Table 1 provides an overview of the top genes that belong to each group of interest.

### 2.3. Inter-Regionally Overlapping Canonical Pathways and Upstream Regulators

Within the FC samples, 184 genes were identified as differentially expressed between the sCJD and control tissues (Supplementary material S1b, a list of the 184 DEGs); whereas, within the CB samples, we identified 88 DEGs (Supplementary material S1c, a list of the 88 DEGs). In total, 68 DEGs were found to be common between FC and CB (Figure 4a; Supplementary material S1d, a list of 68 DEGs).

**Figure 2:**
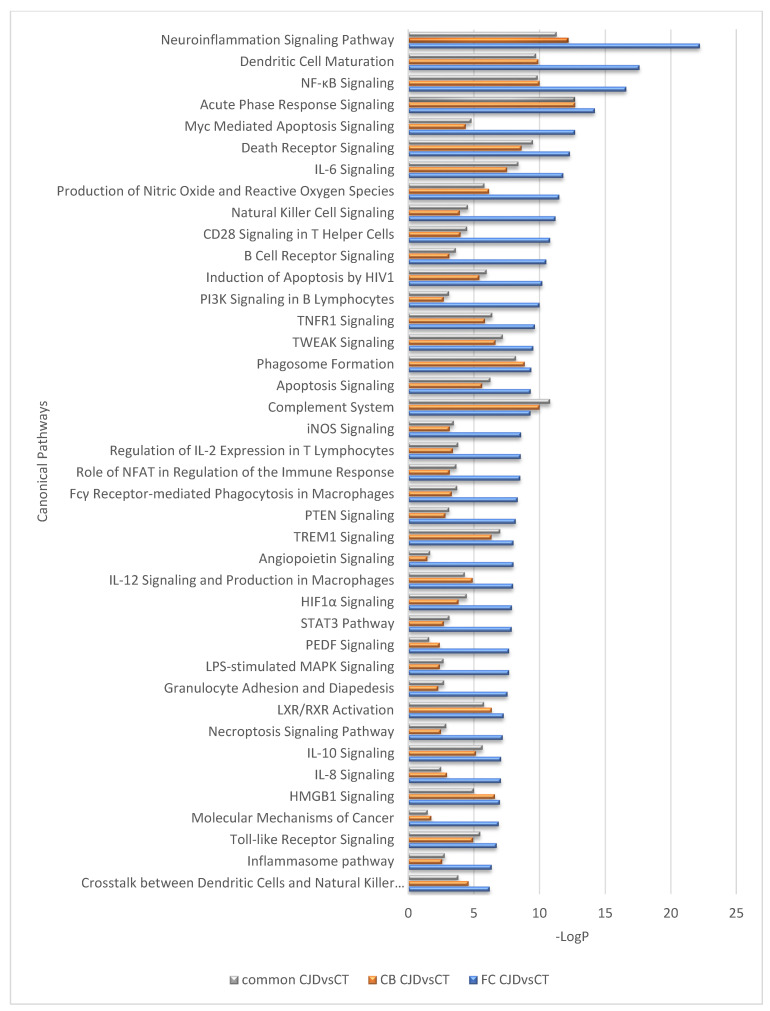
Top 40 canonical pathways regulated differently between the sCJD and control samples but overlapping between the sCJD FC and CB.

**Figure 3:**
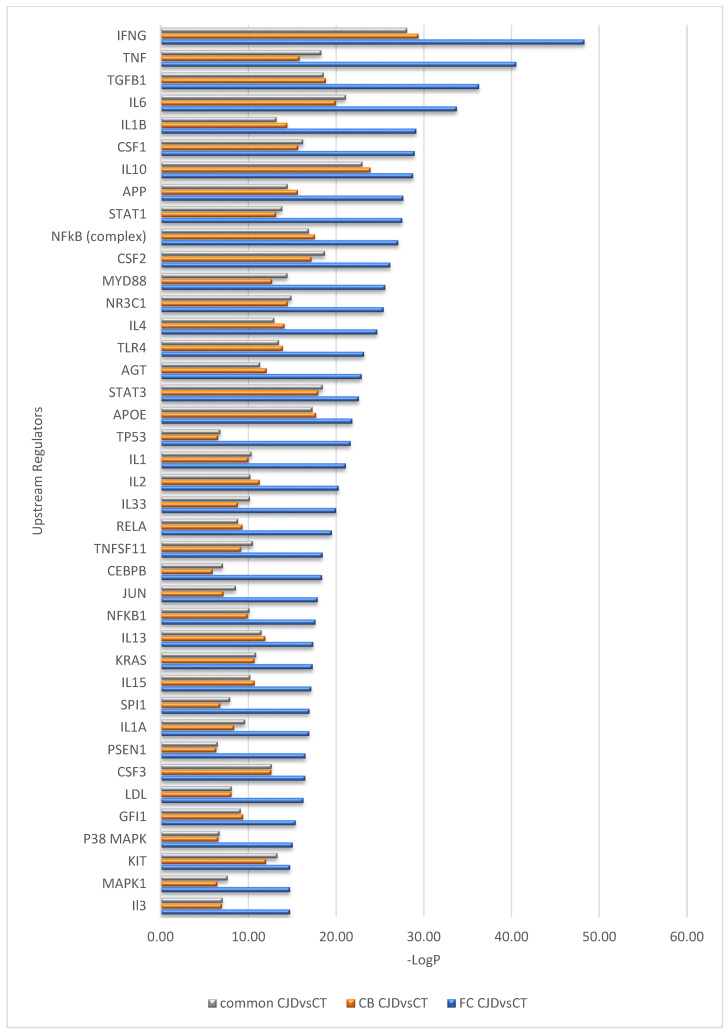
Top 40 upstream regulators predicted differently between the sCJD and control samples but overlapping between the sCJD FC and CB.

Interestingly, despite differences in the number of DEGs identified in the FC and CB sCJD disease signature, the effects of these gene sets appear very similar at the pathway level; a near-complete overlap between canonical pathways for the two contrasts (FC sCJD and CB sCJD) is observed, with the main difference being in the *p*-values, which reflect higher significance for FC due to the higher number of DEGs identified in this tissue. The most significantly enriched canonical pathways shared between FC and CB included the neuroinflammation signaling pathway, dendritic cell maturation, NF-κB signaling, acute phase response signaling, and Myc-mediated apoptosis signaling (Figure 2). An overlap was also observed among upstream regulators identified in the FC and CB sCJD samples when compared to the control samples. The top inter-regionally common upstream regulators included IFNG, TNF, TGFB1, IL-6, and IL-1B (Figure 3). Lists of the top 40 identified pathways and upstream regulators are provided in Figure 2 and Figure 3.

### 2.4. sCJD FC and CB-Exclusive DEGs

**Figure 4:**
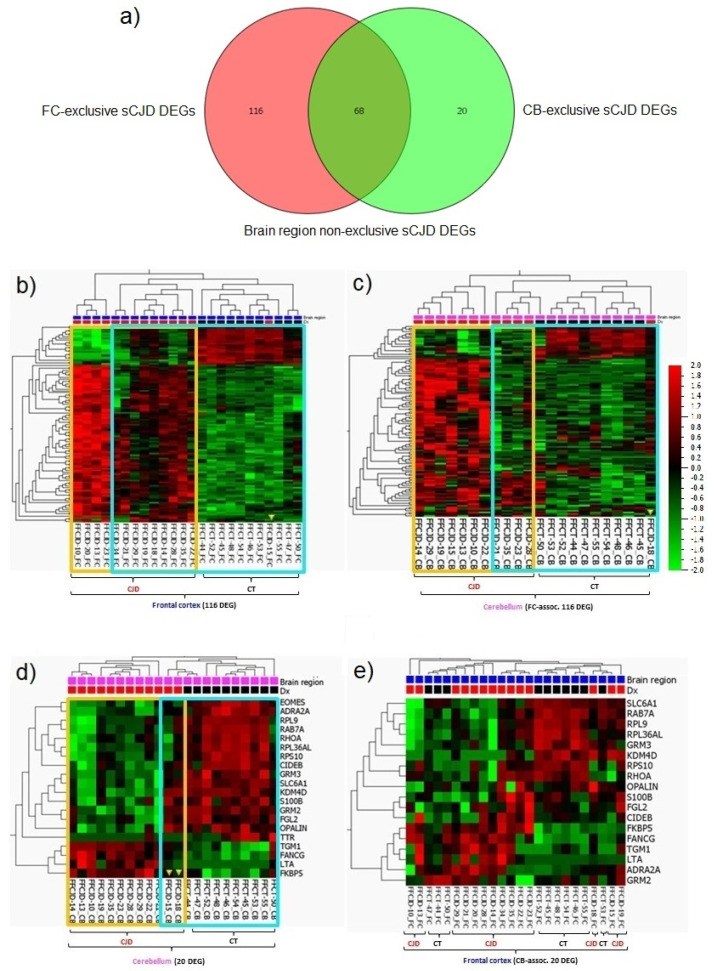
sCJD FC and CB specific gene profiles. (**a**) Graphic number visualization of DEGs that are exclusive and common to sCJD FC and CB samples. (**b**) Clustering of FC sCJD and control samples based on the 116 FC-associated gene signatures. (**c**) Incomplete clustering of the CB sCJD and control samples based on the 116 FC-associated gene signatures. (**d**) Clustering of CB sCJD and control samples based on the 20 CB-associated gene signatures. (**e**) Poor clustering of FC sCJD and control samples based on the 20 CB-associated gene signatures. Yellow frame indicates clusters of the same type samples; blue frame highlights the main clusters indicated by unsupervised hierarchical clustering; yellow arrow heads indicate samples out of their clusters.

Although the FC and CB sCJD signatures shared 68 DEGs, resulting in largely overlapping core molecular processes, each brain region also demonstrated unique single-gene expression patterns. The FC-exclusive-sCJD signature consisted of 116 DEGs, while the CB-exclusive-sCJD signature consisted of only 20 DEGs (Figure 4a; Supplementary material S1e, lists of 116 and 20 DEGs). When the FC-exclusive-sCJD signature was applied to the CB samples, the sCJD and control tissue clusters were formed, confirming that the FC 116 DEGs are sCJD-specific and that their expression profile differs between the two brain regions (Figure 4b,c).

Although the sCJD CB-exclusive signature consisted of only 20 DEGs, they clearly clustered the sCJD and control tissue CB samples apart. Interestingly, when the CB signature was tested on the FC samples, the clustering of the sCJD and control tissue samples was rather poor, confirming that this 20 DEG profile is sCJD CB-specific (Figure 4d,e).

However, when evaluating these results, one should keep in mind that the analysis is based on and biased by a background of 800 pre-selected genes that are mainly neuroinflammatory.

### 2.5. Sub-Regional Differences: Variance in the Strength of Neuroinflammation

**Figure 5:**
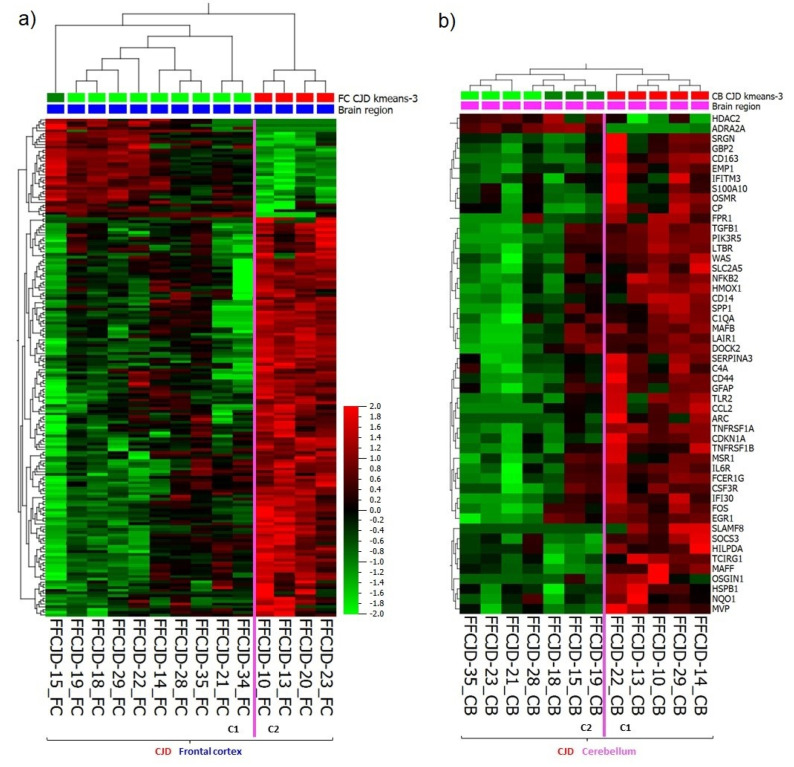
Heatmap and unsupervised two-way hierarchical clustering based on (**a**) 181 DEG between FC sub-cluster 1 (C1) and sub-cluster 2 (C2). (**b**) 50 DEG between CB sub-cluster 1 (C1) and sub-cluster 2 (C2). CJD—sporadic Creutzfeldt–Jakob disease; FC—frontal cortex; CB—cerebellum.

Interestingly, under the sCJD clusters there was a formation of two FC and two CB sub-clusters (Figure 1a). In FC, the gene expression analysis of the first sub-cluster (C1) versus the second sub-cluster (C2) revealed 181 DEGs which overlapped with the FC sCJD versus control tissue DEG signature (Figure 5a) (Supplementary material S1f, a list of the 181 DEGs). In CB, the difference in gene expression pattern between the two sub-clusters was even more apparent because the C2 resembled the gene expression pattern seen in the control tissues, which suggested that some sCJD patients may present with pronounced cerebellar inflammation and some seem to lack neuroinflammatory changes (Figure 1a). The gene expression analysis of the CB C1 versus C2 revealed 50 DEGs (Figure 5b; Supplementary material S1g, a list of the 50 DEGs).

Altogether, analyses of the DEGs involved in the FC and CB sub-clusters formation confirmed variance in the intensity of sub-regional inflammation and the existence of “strong” and “weak” neuroinflammation profiles.

## 3. Discussion

The involvement and dysregulation of major biological processes such as immune system response, metabolism, developmental biology, and vesicle-mediated transport in prion disease pathogenesis have been confirmed by numerous transcriptomic studies using both human and animal tissues [14,15,16,18,19,24,25,26,28,29,30,31]. However, data on the neuroinflammatory events taking place in different brain regions of human sCJD are surprisingly scarce.

Thus, we hypothesized that the expression of neuroinflammation-associated genes is different in distinct brain regions and aimed to identify disease- and brain region-specific genes as well as to provide an overview of the neuroinflammatory landscape in sCJD patients’ brains using an 800 gene expression panel designed by NanoString. To our knowledge, this is the first published study describing the expression of neuroinflammation-associated genes in human brain samples from the FC and CB of sCJD patients and age- and sex-matched normal controls using such a panel.

We generated data that provide an overview of differentially expressed genes, the most involved canonical pathways and upstream regulators, and their biological functions in sCJD patients as compared to controls; in FC compared to CB; and even the sub-clusters observed within each brain region. The data indicated neuroinflammatory differences in distinct brain regions and varying intensities of inflammation within the same brain regions of sCJD patients with different disease subtypes, which broadens our understanding of prion disease pathogenesis from the aspect of inflammation (Table 1).

Interestingly, the regional sub-clusters could not be explained by patients’ sex, age group, polymorphic codon 129 in the *PRNP*, or type of dominant PrP^Sc^. Undoubtedly, a study with a larger number of sCJD cases with different subtypes would ensure firmer conclusions. Nevertheless, the current results imply that the impact of the sCJD subtype may not be the strongest or sole factor determining the strength of the neuroinflammatory gene expression profile.

Curiously, a study by Makarava et al. performed with the NanoString neuroinflammation panel to investigate mice infected with 22L (astrocyte-associated) and ME7 (neuron-associated) PrP^Sc^ strains found that their established signature for prion disease-associated gene expression was independent of the brain region or prion cell tropism [32].

In our study, however, although the neuroinflammation may seem uniform, considering the overlap of canonical pathways and upstream regulators, we still see specific genes, the expression of which is regulated differently in different brain regions. It is important to look for changes at the single gene expression level to ensure that their unique and subtle roles in disease pathogenesis are not overlooked when multiple genes are pooled together in the enriched sets needed for pathway determination.

Other independent research groups using PCR and immunohistochemistry-based gene and protein expression approaches for the investigation of human sCJD brain immunity also identified the presence of regional differences.

For example, Llorens et al. investigated the expression of 25 selected inflammation-associated genes in the FC and CB of 30 sCJD patients with the MM 1 and VV 2 subtypes [25]. They observed that the upregulation of these genes was higher in the FC in sCJD MM 1 and in the CB in sCJD VV 2 and concluded that regional gene regulation differences depend on the patient’s genotype at codon 129 in the *PRNP* [25,26]. Furthermore, Franceschini et al. proposed that the PrP^Sc^ strain and polymorphic codon 129 also influence regional microglia activation, because their study of activated microglia distribution throughout the brain of different sCJD subtypes demonstrated that microgliosis, PrP^Sc^ deposition, and spongiform change were correlated but varied markedly among the sCJD subtypes [11].

Importantly, the studies on human sCJD samples were in agreement with the notion that neuroinflammation in the brain of sCJD patients is not regionally uniform. However, to understand the key cellular and molecular differences between and within distinct brain regions presenting variable pathology, a larger and statistically higher-powered study is needed, preferably combining data on spatial cells’ distribution and their molecular signatures.

Currently, scientific evidence implies that microglia are the key drivers of neuroinflammation in prion disease, and the pathway analysis based on the DEG sets we identified in our study supports this notion, as the most significantly involved pathways include neuroinflammation signaling and metabolic processes [19]. Nevertheless, our pathway analysis also suggests the importance of brain dendritic cells, providing new insight on the different cell types involved in the disease development (Figure 2).

Furthermore, in our study we identified regionally exclusive genes that are likely involved in the formation and development of different brain structures, such as FC and CB. However, we also provided a list of disease-associated top differentially expressed genes in the two brain regions as well as common DEGs. A few literature sources attempting to explain the role of these genes in prion or other neurodegenerative disease pathogenesis were found.

For example, in the brain SERPINA3 is mainly expressed by astrocytes, and its deregulation has been linked to the pathogenesis of several diseases, including schizophrenia and Alzheimer’s [33,34,35]. SERPINA3 overexpression has previously been reported in the frontal and occipital cortices of various human prion diseases [36]. Several hypotheses considering the pathogenic effects of SERPINA3 overexpression in prion diseases have been proposed—e.g., that it may contribute to PrP^Sc^ formation or hamper PrP^Sc^ clearance [36]. However, conclusive explanations for the molecular mechanism of SERPINA3 upregulation and its role in disease development are still lacking.

SOCS3 proteins are expressed by multiple immune cells where they reside in a cell’s cytosol and negatively regulate cytokines that signal through the JAK/STAT pathway. SOCS3 was one of the few identified genes that were suggested to be prion disease pathogenesis-specific [37]. In another RNA expression study with prion-infected mice models, SOCS3 was detectable early and regulated by the phosphorylation of specific STAT protein complexes [38].

FCER1G is a microglia-expressed hub gene associated with aging and neurodegeneration [39]. Changes in the FCER1G expression were also reported in mice infected with PrP^Sc^ [40,41]. The FCER1G is a part of larger molecular complexes and among its other functions is considered to selectively mediate IL-4 production by basophils, to prime T cells towards effector T-helper 2 subset, to form a functional signaling complex in myeloid cells, and to drive the maturation of antigen-presenting cells.

CD44 expression is linked to reactive astrocyte heterogeneity observed in the brain during prion disease in mice, and it was suggested as a biomarker to enhance the identification of distinct prion agent strains [42]. CD44 antigen is a receptor for hyaluronic acid and can interact with SPP1 and other ligands, allowing it to participate in a wide variety of cellular functions, including lymphocyte activation, recirculation and homing, hematopoiesis, and tumor metastasis.

Finally, the *SPP1* encodes a cytokine known for the upregulation of interferon-gamma and interleukin-12 expression and the reduction in interleukin-10 production, leading to type I immunity characterized by intense phagocytic activity. SPP1 is a key factor regulating the degeneration and regeneration of injured nerves via the c-Fos, PKCα, and p-ERK/ERK pathways [43]. Moreover, SPP1 was found to be upregulated and act as an essential modulator of macrophage phenotypes and their ability to clear pathogenic beta-amyloid forms in mice models of Alzheimer’s disease [44,45]. However, its role in prion diseases has not yet been determined.

The biological roles of these and other genes listed in Table 1 seem highly relevant to prion disease pathogenesis and suggest the importance of various brain cells’ proliferation, differentiation, migration, and trafficking characteristics. To elucidate the phenotypic and functional heterogeneity of brain cells in their various pathologic microenvironments observed in distinct brain regions as well as within the same brain region of different human sCJD sub-types, methods that enable the collection of multiplex molecular and spatial information, such as NanoString GeoMx digital spatial profiler or advanced mass spectrometry-based proteomics, could be of great value [46,47]. For the reproduction and research of specific brain microenvironments and cell subtypes within them as well as the investigation of the effectiveness of novel therapeutic compounds for disease treatment or prevention, humanized cerebral organoid platforms and advanced spatial transcriptomic techniques such as multiplexed error-robust fluorescence in situ hybridization could also benefit the future research of prion disease [48,49].

## 4. Materials and Methods

### 4.1. Samples

Fresh frozen (FF) brain samples from two brain regions—namely, the FC and CB—were collected from 14 sCJD patients and 10 brain donors with no neuropathological changes. In total, 47 RNA samples were analyzed: 27 sCJDs and 20 normal controls. One commercial brain RNA sample included in the NanoString nCounter training kit (Lot. 5201A-0618, 80036) was used as a control and a reference sample for potential experiments in the future.

Age- and sex-matched sCJD and healthy brain samples included seven male and female sCJD patients and five male and female healthy brain donors. Median age at death of sCJD patients was 73 years (range 60–77), and the median age at death of the healthy brain donors was 67.5 years (range 57–74). A maximum of 3 years difference was allowed when selecting age- and sex-matched healthy brain donors for sCJD patients.

The included patients had the following sCJD subtypes: 5/14 were sCJD MM 1, 4/14 were VV 2, and 5/14 were single cases of different subtypes, including MV 1, MV 2K, MV 2K + C, MM 1 + 2, and MM 2C + 1.

All the sCJD samples were from the Danish prion diseases cohort gathered at the Danish Reference Center for Prion Diseases. All the control tissue samples were received from Edinburgh Brain Bank, University of Edinburgh, Scotland (Supplementary material S3).

### 4.2. RNA Extraction and Evaluation

Total RNA extraction from FF brain samples was performed using the RNeasy Mini Kit (Qiagen, Hilden, Germany) following the manufacturer’s recommendations. The Bioanalyzer 2100 (Agilent Technologies, Santa Clara, CA, USA) and NanoDrop spectrophotometer (Thermo Fisher Scientific, Waltham, MA, USA) were used to evaluate the extracted RNA purity (wavelength absorbance ratio A260/280 ~2.0, and A260/230 ~2.0), concentration (ng/µL), and quality (DV300 ≥ 50%, the percentage of RNA fragments ≥300 nucleotides) or integrity (RIN > 4).

### 4.3. NanoString

A commercially available NanoString 757 gene neuroinflammation panel complemented with an additional 30 genes of choice and 13 housekeeping genes was used to generate the expression data (Supplementary material S2, a list of genes in the panel). To our knowledge, this new panel with pre-selected genes associated with immunity and inflammation, neurobiology and neuropathology, and metabolism and stress has not previously been used in investigations of human prion diseases.

The total RNA input for each sample extracted from fresh frozen tissues was 50 ng. All the included samples had an A260/280 absorbance ratio between 1.97 and 2.17, and at least 50% of all the RNAs present in each sample were equal to or longer than 300 base pairs; alternatively, the RNA integrity value (RIN) value had to exceed 4.

The nCounter XT CodeSet Gene Expression Panel assay and Prep Station were used for the direct hybridization of unique barcodes to target RNAs and their placement into the cartridges. A Digital Analyzer was used for the detection and the counting of hybridized probes, and nSolver software version 4.0 was used for data normalization. All the procedures were performed following the NanoString guidelines.

Using nSolver Advanced Analysis, the quality of the experiment was assessed by hybridization effectiveness, which is measured by the expression of internal six positive and eight negative RNA control transcripts included in the CodeSet. The same six positive controls were used for the normalization of the run-to-run introduced count variability.

The sample-to-sample variability was normalized using housekeeping genes selected by the geNorm algorithm. In total, 13 housekeeping genes were included in the neuroinflammation panel: AARS, ASB7, CCDC127, CNOT10, CSNK2A2, FAM104A, GUSB, LARS, MTO1, SUPT7L, TADA2B, TBP, XPNPEP1.

### 4.4. Statistical Analysis

The genes that were differentially expressed between groups were identified by ANOVA (*p* < 0.05), and significance was adjusted for multiple testing by estimating false discovery rate (FDR) [50]. For a gene to be considered differentially expressed in a paired analysis, the p value should be lower than 0.05 (*p* < 0.05) and its log2-fold change higher than 1 (log2FC > 1), unless indicated otherwise. All the data were log2-transformed prior to analysis and visualization in Qlucore Omics Explorer v.3.6 (Qlucore AB, Lund, Sweden), including principal component analysis, heat maps, and unsupervised hierarchical clustering. Functional analysis, including pathway, upstream regulator, and network analysis, was performed in Ingenuity Pathway Analysis (IPA, Qiagen, Redwood City, CA, USA).

## 5. Conclusions

The current study presents the results of the first attempt to reveal molecular neuroinflammatory events occurring in different brain regions of sCJD patients using the NanoString 800 gene neuroinflammation panel+. Our preliminary data on regional brain microenvironments indicate distinct neuroinflammation patterns between the different brain regions and within the same brain region, implying the presence of immune cells with different activation statuses and molecular profiles that might be specific to certain co-factors in their immediate microenvironment.

The current study presents an overview of the most involved neuroinflammation-associated pathways and their biological functions affecting different brain regions in sCJD. Furthermore, we identified several significantly deregulated and functionally interesting genes that are of value for further studies.

Future research is needed for an extensive molecular characterization of different cell types in order to recognize their impact on the microenvironment and, hence, disease development. This knowledge is needed to improve the current diagnostic strategies and speed up the discovery of therapeutically targetable molecules.

## Figures and Tables

**Table 1:** Summary of differentially expressed genes, their molecular function, and their associated biological processes.

	Brain Region	Gene	Gene Expression	Molecular Function ^4^	Biological Process ^4^
Group 1 DEGs ^1^	Frontal Cortex & Cerebellum	SERPINA3 ^†^, Serpin Family A Member 3	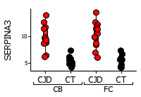	The SERPINA3 protein inhibits serine proteases by binding to them, and thus inducing an irreversible conformational change; identical protein binding.	Acute-phase response; cellular protein metabolic process; endoplasmic reticulum to Golgi vesicle-mediated transport; neutrophil degranulation; post-translational protein modification; blood coagulation.
		SOCS3, Suppressor of Cytokine Signaling 3	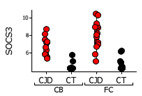	Negatively regulates cytokine signal transduction; 1-phosphatidylinositol-3-kinase regulator activity; phosphotyrosine residue binding; protein kinase inhibitor activity.	Negative regulation of apoptotic process and inflammatory response, of receptor signaling pathway via JAK-STAT, and of tyrosine phosphorylation of STAT protein; positive regulation of cell differentiation; post-translational protein modification.
		SPP1 ^†^, Secreted Phosphoprotein 1	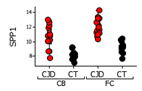	Probably important to cell-matrix interaction; cytokine activity; extracellular matrix binding; integrin binding.	Cell adhesion; cellular protein metabolic process; inflammatory response; positive regulation of transcription, DNA-templated; post-translational protein modification.
		CD44 ^†^, CD44 Molecule	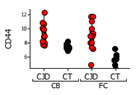	Cell-surface receptor that plays a role in cell-cell interactions, cell adhesion and migration, helping them to sense and respond to changes in the tissue microenvironment; collagen and hyaluronic acid binding.	Positive regulation of heterotypic cell-cell adhesion; cell migration; extracellular matrix disassembly; negative regulation of apoptotic process; inflammatory response.
		FCER1G, Fc Fragment of IgE Receptor Ig	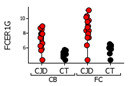	IgE-binding protein; receptor; identical protein binding; IgG binding.	Immunity; innate immunity; mast cell activation; phagocytosis, engulfment; positive regulation of interleukin-10, -6, and -4 production.
Group 2 DEGs ^2^	Frontal Cortex	DAB2, DAB Adaptor Protein 2	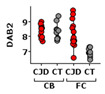	Adapter protein; cargo receptor activity; clathrin adaptor activity; protein C-terminus binding; SMAD binding.	Apoptotic process; cell differentiation; membrane organization; protein transport; negative regulation of protein binding and protein localization to plasma membrane; positive regulation of cell migration and early endosome to late endosome transport.
		ITGB5, Integrin Subunit Beta 5	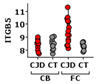	A receptor for fibronectin; integrin binding; signaling receptor activity; virus receptor activity.	Cell adhesion mediated by integrin; cell migration; extracellular matrix organization; stress fiber assembly; host-virus interaction.
		GRAP *, Growth Factor Receptor-Bound Protein 2-Related Adaptor Protein	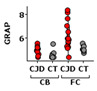	Couples signals from receptor and cytoplasmic tyrosine kinases to the Ras signaling pathway.	Cell-cell signaling; Ras protein signal transduction; sensory perception of sound.
		TGFBR1, Transforming Growth Factor Beta Receptor 1	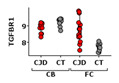	Serine/threonine-protein kinase; receptor; activin binding; ATP binding; metal ion binding; SMAD binding; transforming growth factor beta binding.	Apoptosis; differentiation; growth regulation.
		TNFRSF10B *, Tumor Necrosis Factor Receptor Superfamily, Member 10b	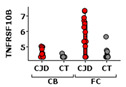	Receptor for the cytotoxic ligand TNFSF10/TRAIL mediating apoptosis; promotes the activation of NF-kappa-B; essential for ER stress-induced apoptosis.	Apoptosis; cellular response to mechanical stimulus; leukocyte migration; response to endoplasmic reticulum stress.
	Cerebellum	ADRA2A *, Adrenoceptor Alpha 2A	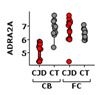	G-protein coupled receptor; transducer; protein heterodimerization activity; protein homodimerization activity.	Cellular response to hormone stimulus; glucose homeostasis; positive regulation of cell migration, cell population proliferation, cytokine production; Rho protein signal transduction.
		GRM2, Glutamate Metabotropic Receptor 2	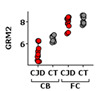	G-protein coupled receptor; transducer; may mediate suppression of neurotransmission or may be involved in synaptogenesis or synaptic stabilization.	Chemical synaptic transmission; glutamate homeostasis and secretion; G protein-coupled glutamate receptor signaling pathway; regulation of synaptic transmission, glutamatergic.
		RHOA, Ras Homolog Family Member A	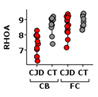	Hydrolase; in neurons, involved in the inhibition of the initial spine growth. Upon activation by CaMKII, modulates dendritic spine structural plasticity.	Cell cycle; cell division; host-virus interaction; cerebral cortex cell migration; cellular response to chemokine and cytokine stimuli; forebrain radial glial cell differentiation; neuron apoptosis, morphogenesis, proliferation, and differentiation.
Group 2 DEGs ^2^	Cerebellum	S100B, S100 Calcium Binding Protein B	* 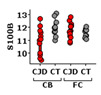 *	Calcium-dependent protein binding; identical protein binding; protein homodimerization activity; tau protein binding.	Astrocyte differentiation; central nervous system development; innate immune response; regulation of cell shape; positive regulation of cell population proliferation and apoptotic process.
		SLC6A1, Solute Carrier Family 6 Member 1	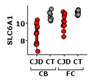	Terminates the action of GABA by its high affinity sodium-dependent reuptake into presynaptic terminals; identical protein binding; metal ion binding; neurotransmitter binding.	Neurotransmitter transport; neurotransmitter reuptake; synapse organization; transport across blood-brain barrier.
Group 3 DEGs ^3^	Frontal Cortex	ASB2, Ankyrin Repeat and SOCS Box Containing 2	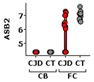	Mediates the ubiquitination and subsequent proteasomal degradation of target proteins.	Intracellular signal transduction; post-translational protein modification.
		DLX1, Distal-Less Homeobox 1	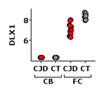	Transcriptional activator or repressor; plays a role in differentiation of interneurons, in the development of the ventral forebrain and diencephalic subdivisions, in craniofacial patterning and morphogenesis.	Cell differentiation; transcription; transcription regulation; developmental protein.
		NRGN, Neurogranin	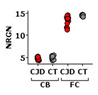	Acts as a messenger during synaptic development and remodeling; calmodulin binding; phosphatidic acid binding; phosphatidylinositol-3,4,5-trisphosphate binding.	Nervous system development; positive regulation of long-term synaptic potentiation; postsynaptic modulation of chemical synaptic transmission; signal transduction; telencephalon development.
	Cerebellum	EOMES, Eomesodermin	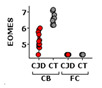	Transcriptional activator; plays a role in brain development being required for the specification and the proliferation of the intermediate progenitor cells and their progeny in the cerebral cortex.	Adaptive immune response; brain development; cell fate specification; cerebral cortex neuron differentiation; cerebral cortex regionalization; stem cell population maintenance; interferon-gamma production.
		TTR, Transthyretin	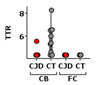	Probably transports thyroxine from the bloodstream to the brain; hormone activity; identical protein binding; protein-containing complex binding; thyroid hormone binding.	Cellular protein metabolic process; extracellular matrix organization; neutrophil degranulation; purine nucleobase metabolic process.

^1^ Disease-specific, brain region non-exclusive differentially expressed genes (DEGs). ^2^ Disease-specific, brain region exclusive DEGs. ^3^ Disease non-specific, brain region exclusive DEGs. ^4^ Information gathered from http://uniprot.org. * Gene, the expression of which is highly differential also between the sub-clusters of the given sCJD brain region. ^†^ Gene, the expression of which is highly differential also between the sub-clusters of the sCJD cerebellum, but not frontal cortex samples.

## Data Availability

Datasets related to this article are available in the Gene Expression Omnibus (GEO, https://www.ncbi.nlm.nih.gov/geo/) with the following accession number: (GSE160208) [NCBI tracking system #21381188].

## Supplementary Materials

Supplementary materials can be found at https://www.mdpi.com/1422-0067/22/1/140/s1.

## Abbreviations

sCJD	Sporadic Creutzfeldt–Jakob disease
CT	Control tissue (normal brain)
FC	Frontal cortex
CB	Cerebellum
DEG(s)	Differentially expressed gene(s)
