# Supplementary material for: Regional Differences in Neuroinflammation-Associated Gene Expression in the Brain of Sporadic Creutzfeldt–Jakob Disease Patients
Source: Int J Mol Sci. 2020 Dec 25;22(1):140. doi: 10.3390/ijms22010140 (PMC7795938; doi:10.3390/ijms22010140)
Supplement: Supplementary file 1 [file ijms-22-00140-s001.zip › S3_Control tissues_20_11_2020.docx]

All control tissue samples were received from Edinburgh Brain Bank, funded by the UK Medical Research Council, University of Edinburgh, Scotland. The received samples had the following BBN identifications: BBN_7626, BBN_2654, BBN001.30147, BBN001.30916, BBN001.30208, BBN_2441, BBN001.31504, BBN_2521, BBN001.29882, BBN_14395.
